# Clinical and functional results of radial club hand with centralization and pollicization using the second metacarpus: A clinical case series

**DOI:** 10.1016/j.ijscr.2019.07.076

**Published:** 2019-08-01

**Authors:** Farivar A. Lahiji, Farhang Asgari, Fateme Mirzaee, Zohreh Zafarani, Hamidreza Aslani

**Affiliations:** a Shahid Beheshti University of Medical Sciences, Iran; b Lorestan University of Medical Science, Iran; c University of Social Welfare And Rehabilitation Sciences, Knee and Sport Medicine Research Center, Milad Hospital, Tehran, Iran; d Knee and Sport Medicine Research Center, Milad Hospital, Tehran, Iran

**Keywords:** Radial club hand, Centralization, Pollicization, Metacarpal bone

## Abstract

**Introduction:**

Radial club hand (RCH) is a rare congenital deformity leading in several functional and psychological problems. However, our knowledge about the long-term functional outcomes of treating RCH is limited. In current study, we investigated the outcomes of centralization and pollicization using second or third metacarpal bone in RCH patients.

**Methods:**

There were 15 hands (13 patients) with RCH underwent centralization and pollicization using second or third metacarpal bone or tendon transfer. The patients aged 1.2 ± 1 years at the time of the surgery. On early postoperative x-rays, the forearm-hand angle was measured. The patients were followed for 6.2 ± 2.3 years. At the final visit, disabilities of arm, shoulder and hand (DASH) score was completed. Furthermore, forearm-hand angle and range of motion of both wrists in sagittal and coronal planes were measured.

**Results:**

The mean of forearm-hand angle increased significantly. In 11 wrists, forearm-hand angle increased only 10 degrees of less. The range of operated wrist was improved in sagittal and coronal planes. The relative range of wrist motion in patients with unilateral deformity in sagittal and coronal planes was 83 ± 11 percent and 61 ± 12 percent. Three patients developed skin necrosis.

**Conclusion:**

Early centralization and pollicization using second or third metacarpal bone can significantly restore the range of motion and function in patients with RCH.

## Highlights

• Radial club hand (RCH) is a rare congenital deformity leading in several functional and psychological problems.
• Treatment of RCH should begin as soon as possible after birth.
• Treatment of patients with RCH by primary traction and centralization and pollicization surgery, can greatly improve the deformity.
• Early centralization and pollicization can significantly restore the range of motion and function in patients with RCH.

## 1 Introduction

Radial club hand (RCH) or radial dysplasia is a complex congenital difference occurring in a longitudinal direction resulting in radial deviation of the wrist and shortening of the forearm. In this condition, hand radially deviated at the distal forearm in the shape of a golf club [[1], [2], [3]]. Bone disorders are the most important part of RCH, but abnormalities of muscles, arteries, nerves and joints can greatly affect the function and treatment of upper limb [4]. The reason of naming RCH is that all the radial column of forearm are hypoplastic and as a result these children are often deprived of having a functional thumb and may in the future require pollicization [4,5].

The first newly-born male infant case with bilateral club hand due to total lack of the radius was reported in 1733 by Petit [6]. This deformity is an uncommon condition and its prevalence is estimated at 1:20000 to 1:100000 live births and slightly higher in boys, at 3:2 [1,7,8]. This anomaly is bilateral and asymmetric in 38%–58% of cases [9,10]. Children with bilateral and severe RCH have considerable functional limitations due to thumb and wrist dysfunction, and short upper limbs [1].

Although RCH has a high range of phenotypes from hypoplasia of the thumb to complete lack of the radius and the first ray, Bayne et al. divided this deficiency into four types based on the radiographic severity of the radial ray deficiency [11]: Type I, the mildest type, is determined by radius shortening. People with hypoplastic radius are considered as type II, partial radial absence as type III, and complete absence of radius as type IV. RCH treatment can be highly variable and different based on the patient's age and severity of the deformity. Treatment goals usually involve creating a functional and centralized hand, maintaining the range of motion, stability of the wrist, and sustaining longitudinal growth of forearm [12,13].

Treatment of RCH should begin as soon as possible after birth. Type I and II, usually respond well to stretching and splinting, which will stretch the tight soft tissues and radial structures and allows passive modification of the malformation by aligning the hand and wrist with the ulna [1]. Types III and IV are considered as the most common forms of deformity that are usually associated with the greatest amount of radial deviation of the wrist [4,14]. The most common method of treatment is centralization of the wrist, in which patient’s wrist will be moved to the central part of the distal ulna and its main objective is correction of semi-subluxation and radial deviation, which may unfortunately have a high rate of recurrence of deformity, damaging ulna’s physis, and wrist stiffness [4,9,15].

One of the most important steps is creating a thumb with a good function for the patients, which depending on the severity of thumb anomalies is performed by various techniques such as tendon transfer (in cases of nonfunctional thumb) and pollicization by index finger or the second finger of the foot (in cases without thumb) and some studies have provided relatively good results of this procedure [[14], [15], [16], [17], [18], [19], [20]]. Despite various studies related to introduction of the treatment methods of RCH and evaluation of the results, limited data is available on the long-term outcomes of different treatment methods. The present retrospective study aimed to evaluate the long-term treatment outcomes for RCH patients with correction of deformity with centralization and pollicization using the second metacarpus.

## 2 Materials and methods

The present case series of 13 patients with RCH was conducted in two hospitals from 2006 to2016, on patients undergoing two-staged centralization and pollicization surgery with the second or third metacarpus or tendon transfer who were recruited after signing the written informed consent. Three patients had bilateral RCH and a total of 16 deformed limbs entered the first phase of the study. In one patient with bilateral disease, RCH was type I that did not require surgery and was excluded from the study. In total, the study was performed on 15 limbs in 13 patients with RCH.

Firstly, the charts of all patients evaluated and patient's RCH data were extracted. Then, demographic information, including sex, side of the disease, age at first and second surgery, type of surgery, type of RCH based on Bayne category, type of thumb anomaly according to Buck-Gramcko category, and comorbidities were recorded. Informed consent was obtained from all parents. According to the radiographs taken immediately after the surgery, forearm-hand angle was measured as the angle between the longitudinal axis of ulna and the longitudinal axis of the third metacarpus. Our surgical technique was the same as what has been described by Buck-Gramcko for policization.

**Fig. 1:**
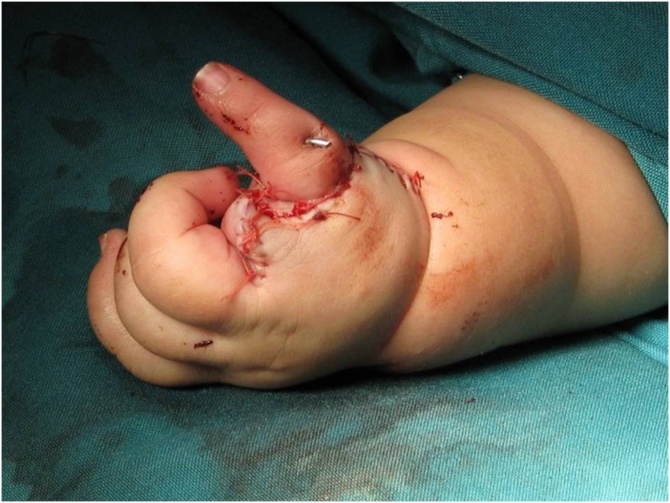
Pollicization with Buck-Gramcko technique.

After diagnosis was established, serial splinting was performed for stretching and patients were referred to a neonatologist for rule out comorbidities that is contraindications for surgery. In the first stage of operative management the radialization or centralization was considered. In most of the cases the ages of the patients were between 6 to 18 months at time of the first stage of operation. The patient was placed in supine position. Under general anesthesia, surgery was performed using bilobed flap approach of Evans. A transverse incision was started on the radial side of the wrist, extending to ulna at an angle of approximately 90°. The flap was elevated, while dorsal veins and sensory nerves were preserved. Radial side incisions were very superficial to prevent damage to the median nerve. The extensor retinaculum was cut in two radial and ulnar sides. Extensor and flexor structures were determined by drawing tendon or muscle mass. If brachioradialis, FCR,^1^ ECRL^2^ and ECRB^3^ existed, they were separated from the insertion. Long digit extensors were retracted to the radial side with special care to maintain the ulnar artery. Dorsal and palmar capsule were cut transversely to release carpoulnar joint. Any fibrotic and contracted structure was excised. Then, the hand was transposed to the ulnar side and wrist’s bones were placed on the ulnar head and a pin was placed from the second metacarpus in radialization or third in centralization into the ulnar bone. Ulnar side of the wrist was reinforced using shortening and tightening of ECU^4^ muscle. Then with 90° rotation, the ulnar and then dorsal flap were covered on the radial side of the wrist and sutured with absorbable 4-0sutures. Then, the hand was placed in a long cast for 6 to 8 weeks. Afterward, the pins removed and short arm splint were made for the patient for full-time use for 6 months. After 6 months the splint should be used overnight until skeletal maturity. If the second operation was necessary for pollicization, Buck –Gramcko technique with some minor modifications was done. We tried to do the policization before age of two (Fig. 1).

The patients were called and asked to come to hospital clinic; if they wished to cooperate in the study, and shoulder and hand score (DASH)^5^ questionnaire was used to assess their functional status of disabilities of arm. This score ranges from zero to 100, and represent the effectiveness of the current situation of the upper limb (unilateral or bilateral) on the ability to perform everyday tasks as a percentage. On this scale, score closer to 100 means greater impact of the upper extremities disease on the individual’s function. In the final visit, anterior-posterior and lateral radiographs were obtained and forearm-hand angle was recalculated and compared with the radiographs taken immediately after surgery. The range of motion of both wrists was measured in the sagittal and coronal planes using a goniometer. In cases where the patient had unilateral deformity, wrist’s range of motion was compared, relative to the normal wrist. Quantitative data were presented as mean ± SD^6^ and qualitative data as numbers and percentages. To check the data and for statistical analysis, SPSS^7^ ver.16 software was used. The Wilcoxon test was used to compare forearm-hand angle. P < 0.05 was considered as the significance level.

This the research work has been reported in line with the PROCESS criteria [21].

## 3 Results

As stated, 15 limbs in 13 patients with RCH were examined. It should be noted that among three patients with bilateral RCH, one limb was excluded from the study due to response to non operative treatment. The patients' demographic informations are provided in Table 1. The table shows, the distribution of genders was almost equal. In the first stage of centralization surgery, rotational flap and wrist tendon transfer were applied for all patients. Due to the abnormality of thumb, tendon transfer of thumb was required in 4 hands and pollicization in 6 patients, but due to the lost to follow-up, tendon transfer of thumb was performed in 3 patients and pollicization in 4 patients.

**Table 1:** Demographic and background information of the patient.

**Number of patients**	13		
**Number of limbs**	15		
**Age(at the time of first surgery, year)**	1.2±1 (1_1.5)		
**Duration of the follow up**	6.2±2.3 (1_9)		
**Gender**	M		F
**(M/F)**	6(46.1%)		7(53.9%)
**Secondary procedure**	Pollicization 4		Wrist Tendon transfer 3
**Radial club hand type**	II 1(6.6%)	III 7(46.7%)	IV 7(45.7%)

Mean forearm-hand angle in Radiography taken immediately after surgery was 13.8 ± 5° (range: 10–23) that significantly increased in the final visit to 22.2 ± 13.5° (10–60) (P = 0.005). It should be said that in 11 hands, the increase in forearm-hand angle was negligible (≤10°). The mean DASH score in the final visit was 34.2 ± 9.7 (range: 18–55) (Table 2). The mean DASH score in 4 patients with pollicization were 39.3 ± 12 (range: 28.1–55.2).

**Table 2:** Comparison of outcomes immediately after surgery and final visit.

	Immediately after surgery	Final visit	P_value
**Sagittal ROM(º)**	90.92±21.48 (20_140)	103±32 (20_140)	0.221
**Coronal ROM(º)**	21.92±8.47 (10_40)	25±8 (10_40)	0.556
**Forearm hand angle(º)**	13.8±5 (10_23)	22.2±13.5 (10_60)	0.005
**DASH score**	44.38±11.42 (20_60)	34.2±9.7 (18_55)	0.069

ROM: range of motion.

Range of motion in the operated wrist in sagittal and coronal planes was 103 ± 32° (20–140) and 24 ± 8 (10–40). In fact, in 4 hands, limitation of motion in the sagittal plane was very severe and range of motion was less than 80°. In these cases, the range of motion in the coronal plane was much more restricted than others.

We compared range of motion of normal and operated wrists in patients with unilateral deformities. The results of the comparison are presented in Table 3. The relative range of motion of the wrist in the sagittal and coronal plane in the operated hand was 83 ± 11% (range: 55–96) of the normal hand and in the coronal plane was 61 ± 12% (range: 40–78%). Skin necrosis occurred in three patients.

**Table 3:** Comparison of sound wrist and underwent surgery wrist outcomes in unilateral patients.

variables	sound wrist	underwent surgery wrist	P_value
**Sagittal ROM(º)**	140±6 (130_150)	117±17 (75_140)	0.003
**Coronal ROM(º)**	47±5 (40_55)	28±6 (20_40)	0.001˂

ROM: range of motion.

## 4 Discussion

The most important finding of this study was that two-step treatment of RCH, including centralization with rotational flap and wrist tendon transfer and subsequently attempts to create a functional thumb with pollicization using the second or third Ray or tendon transfer that is associated with favorable clinical outcomes and function in most patients.

RCH is a longitudinal defect that can cause a wide range of upper extremity deformities and its etiology may be sporadic, unknown, or associated with a clinical syndrome like Holt-Oram syndrome, Thrombocytopenia-Absent Radius (TAR), VACTERL and Fanconi anemia [4]. This disease can have a wide range from a mild illness with nonsurgical treatment to severe disease with multi-staged surgical treatment. In this disease, the radial column of forearm is hypoplastic, thus the thumb is often not functional and, if untreated, the disability will remain. On the other hand, because of the low incidence of this deformity, the numbers of relevant studies are limited and are mostly case series. So, we have very little information and appropriate treatment and prognosis is unclear to us. Due to problems associated with these patients, regardless of the severity of anomaly, initial assessment of general health status such as heart, kidney, gastrointestinal and blood disease, is necessary. Therefore, electrocardiography, renal ultrasound, blood analysis and chromosomal breakage must be performed for all patients.

In previous studies, different surgical and nonsurgical treatment methods have been proposed. The patient is candidate of serial splinting and stretching, aimed to maintain passive correction of wrist deformity [4,12]. Absolute contra-indications for surgery include the following:

1 Older kids with established disease
2 Associated medicals anomalies
3 Elbow contracture in extension
4 Mild deformity with acceptable cosmetic status and function.

The goal of surgical treatment is to achieve the desired length of the upper limb, correction of forearm deviation, reconstruction of thumb, and pollicization. Wrist realignment includes centralization and radialization with similar surgical exposure in both methods. In radialization, it was tried that the radial ulnar deformity be overcorrected to align ulna with the index finger, and all the force of extrinsic muscles (flexor carpi radialis, brachioradialis, extensor carpi radialis longus and brevis) move to the ulnar side. In centralization, ulnar axis is aligned with the third metacarpus.

Regarding the thumb status, it must be said that in hypoplasia grade I, there is often do not need surgery. In hypoplasia grade II, treatment includes opponens plasty, fixation of metacarpophalangeal joint and 1^st^ web Deeping. In severe degrees of grade III, IV and V, usually pollicization is used to improve pinch and grasp status. Other methods of treatment in hypoplasia grades IV and V include microvascular joint transfer that uses the second toe of the left foot. In 1998, Vilkki et al, introduced this method and reported favorable results in 9 patients with RCH grade IV [20]. In 2008, Vilkki and his colleagues increased the number of patients and reported the treatment outcomes of 19 hands in 18 patients. The researchers found that at the final visit, mean forearm-hand angle was 28° of radial deviation, mean range of motion of the wrist joint was 83°, mean ulnar growth was 15.4 cm, and mean relative length of ulna, compared to the other side, was 67% [14].

Some scholars, in addition to favorable treatment results, have evaluated the recurrence rate of deformity in RCH following centralization and have achieved significant results. Shariat Zadeh and colleagues examined the recurrence of deformity after centralization. In this study, 11 hands underwent centralization. Patients were followed for 90 months. Mean pre-surgical hand-forearm angle was 75° that reached 25° immediately after surgery and 52° in the final visit. Thus, the correction rate was 66% and the rate of loss of correction was 54%. However, the researchers did not state how many patients had a recurrence of deformity [15]. Damore and colleagues treated 19 cases of RCH with centralization in 2000 and observed that angulation declined from 83° to 25° immediately after the surgery (58° improvement). However, in the final visit after 3.3 years, it reached 63° (38° loss of correction) [22]. Lamb and his colleagues also said 7 patients had recurrence of deformity (46.7%) [23]. We had recurrence in 4 hands (26.7%) that seems acceptable and is lower than other studies, compared with previous studies.

Another study in 2008 by Kanojia and his colleagues examined the outcomes of RCH type III and IV in 18 hands. In this study, soft tissue distraction before centralization and transfer of flexor carpi radialis and flexor carpi ulnaris tendons to the fifth finger was performed. The mean age at the onset of treatment was 8 months. In 16 cases, treatment was completed before 10 months of age. In this study, before centralization, soft tissues were stretched sufficiently and the results after 31 months after surgery was good in 7 patients, satisfactory in 8 cases, and unsatisfactory in one case [24]. It should be noted that we also used stretching and serial splinting treatment of patients six months before surgery, however, our results seemed weaker, compared to the results of Kanojia’s study. It is likely that better short-term follow-up of study was one of the reasons for this difference.

Like this study, Fujiwara and his colleagues presented a two-step method for treating RCH that corrected angulation and prevented necrosis of the renovated thumb. In this method, the surgeon used centralization with s-shape incision on the dorsum of the hand and one-third of distal forearm. Then FCR and ECR were released and ECU was retracted to the distal side. FCU^8^ was transferred to ECU and wrists were fixed by a 1.8-mm pin. Wound was closed using rotational flap and limbs were immobilized in a long cast for 4 weeks. In the second stage, the index finger was used for pollicization at 31 months of age that was carried out without removal of the primary pin. If the pin does not break, it was drawn 14 months after pollcization. In this study, 4 patients were treated and no recurrent deformity was observed after four years and the results were excellent. Just because of long-term maintenance of pins, there was a risk of breaking pins and pin tract infection [25]. It should be noted that the reason of no cases of recurrence in this study could be due to the limited number of patients. In addition, long-term preservation of pin can also greatly help prevent the recurrence of deformity, but it is so painful and increases the risk of infection. Fujiwara and his colleagues also did not mention to any stiffness of the joint.

Paley et al in 2008 reported on 21 hands in 14 patients who underwent ulnarization between 2000 and 2006. The age of patients ranged from one to 14 years (mean 6 years.) with 46 months follow up. Wrist dorsiflexion (passive), arc of motion and flexion contracture, hand-to-forearm angle and position, palmar carpus displacement, ulnar length improved after Paley ulnarization. Recurrence of deformity, skin necrosis and growth arrest of the ulnar epiphysis was not observed. Overgrowth of the distal ulna relative to the carpus and excessive ulnar deviation reported as the biggest problems of ulnarization. To prevent this complication, Paley recommended to reduce distraction force on the ulnar head by ulnar shortening [26].

Romana, C. treated 13 patients mean age of 37.5 by distraction of mini rail external fixator. This method provided sufficient distraction in the concavity of the deformity to allow satisfactory correction in all cases. Mean distraction time was 53.2 days and ulnar osteotomy was required in 8 cases (61%). They improved sagittal and coronal correction after centralization [27].

Manske, M. C. et al in 2014 recorded a study to evaluate the effect of soft tissue distraction on recurrence of deformity after centralization for radial longitudinal deficienc. 13 upper limbs treated with centralization alone were compared with 13 treated with ring external fixator distraction followed by centralization with 2–10 years follow up. They observed Centralization, with or without distraction with an external fixator, corrected alignment of the wrist. Distraction facilitated centralization, but it did not avoid deformity recurrence and was associated with a worse radial deviation and volar subluxation position compared with wrists treated with centralization alone [28].

**Fig. 2:**
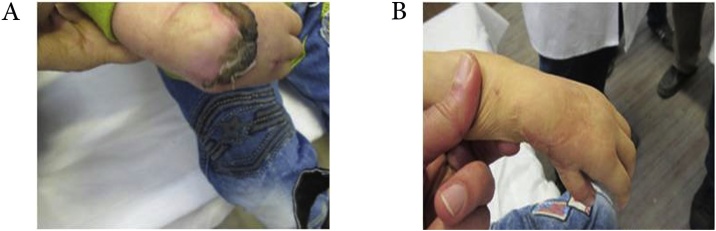
A) Flap necrosis B) healing of the necrotic area.

In our study, we used a two-step method for treatment of RCH. As mentioned, before surgery, soft tissue stretching, and serial splinting was done. At the beginning, patients were evaluated for comorbidities. After splinting, the first surgery of wrist realignment (centralization) and wrist tendon transfer were done. At this stage, rotational flap was used to close the wound, which was in three cases with minor flap edge necrosis no need for surgical intervention (Fig. 2). Then, in the second surgery for patients with abnormalities grade II and III, tendon transfer was used to promote thumb’s function. In cases without thumb (grades IV and V), the second thumb was used for pollicization, except one case that was performed younger than 2 years of age. Our study is one of the few studies that have examined the long-term results of RCH treatment. We observed a deformity recurrence rate of >20° in 4 patients (26.7%) that was not significant in other cases. Range of motion in sagittal and coronal planes, was acceptable in operated hand compared with normal hand that provided the ability to perform daily activities to some extent. Of course, the range of motion in the sagittal plane was better than that of coronal that is fully justifiable regarding the radial deviation of wrist’s deformity. To evaluate the treatment outcomes and impact of residual deformity on person’s function, we used DASH score and observed that this deformity affected the ability of individuals to a considerable extent to perform everyday tasks. In fact, according to our results, residual deformity disrupted about 34% of the person’s function. However, it should be noted that, if untreated, this effect significantly increased. Another point that should be noted here is that in the treatment of RCH, patient’s cooperation and his family is also very important. In our study, three patients required thumb surgery, did not return, which may be one of the reasons of the impact on function outcomes in this study.

Like all other studies, this study had also limitations. One of the most important limitations of this study was to compare the pre- and post-treatment results without control group. Also, the number of patients was not sufficient, and patients were followed shortly. Thus, it seems necessary that further studies evaluate more patients with longer follow-up.

## 5 Conclusion

Treatment of patients with RCH by primary traction and centralization and pollicization surgery, using the second or third metacarpus or tendon transfer can greatly improve the deformity, to gain range of motion and acceptable functional ability. However, this method has its own limitations and is not followed by the desired results, in some cases. Further studies with long term follow-up are required in the future.

## Funding

This research did not receive any specific grant from funding agencies in the public, commercial, or not-for-profit sectors.

## Ethical approval

The ethical approval for the publication of this case series was exempted by our institution because all of the data were collected from clinical records and imaging systems for routine perioperative planning.

## Consent

Written informed consent was obtained from all of the patient's fathers as they are minors, for publication of this case report and accompanying images. A copy of the written consent is available for review by the Editor-in-Chief of this journal on request.

## Author contribution

1-Author name: Farivar A. Lahiji.

Contribution (Type): Therapist.

2-Author name: Farhang Asgari.

Contribution (Type): Therapist.

3-Author name: Fateme Mirzaee.

Contribution (Type): Writer.

4- Author name: Zohreh Zafarani.

Contribution (Type): Editing the manuscript.

5-Author name: Hamidreza Aslani.

Contribution (Type): Editing the manuscript & Corresponding author.

## Registration of research studies

Study registered with Iranian Registry of Clinical Trials 40883.

## Guarantor

Hamidreza Aslani.

## Provenance and peer review

Not commissioned, externally peer reviewed.

## Declaration of Competing Interest

The authors declare no conflicts of interest. The authors have no financial, consultative, institutional and other relationships that might lead to bias or conflict of interest.

## Footnotes

1 Flexor Carpi Radialis.

2 Extensor Carpi Radialis Longus.

3 Extensor Carpi Radialis Brevis.

4 Extensor Carpi Ulnaris.

5 Disabilities of arm shoulder and hand.

6 Standard Deviation.

7 Statistical Package for the Social Sciences.

8 Flexor Carpi Ulnaris.
